# Decision regret in men living with and beyond nonmetastatic prostate cancer in the United Kingdom: A population‐based patient‐reported outcome study

**DOI:** 10.1002/pon.5362

**Published:** 2020-02-26

**Authors:** Sarah Wilding, Amy Downing, Peter Selby, William Cross, Penny Wright, Eila K. Watson, Richard Wagland, Paul Kind, David W. Donnelly, Luke Hounsome, Rebecca Mottram, Majorie Allen, Therese Kearney, Hugh Butcher, Anna Gavin, Adam Glaser

**Affiliations:** ^1^ Leeds Institute of Medical Research at St James's University of Leeds Leeds UK; ^2^ Leeds Institute for Data Analytics University of Leeds Leeds UK; ^3^ School of Psychology University of Leeds Leeds UK; ^4^ Leeds Teaching Hospitals NHS Trust University of Leeds Leeds UK; ^5^ Oxford Institute of Nursing, Midwifery and Allied Health Research Oxford Brookes University Oxford UK; ^6^ Faculty of Health Sciences University of Southampton Southampton UK; ^7^ Academic Unit of Health Economics University of Leeds Leeds UK; ^8^ Northern Ireland Cancer Registry Queen's University Belfast Belfast UK; ^9^ National Cancer Registration and Analysis Service Public Health England Bristol UK

**Keywords:** cancer, decision regret, involvement in decision‐making, LAPCD, oncology, patient‐reported outcomes, prostate cancer, treatment decision‐making

## Abstract

**Objective:**

Clinical options for managing nonmetastatic prostate cancer (PCa) vary. Each option has side effects associated with it, leading to difficulty in decision‐making. This study aimed to assess the relationship between patient involvement in treatment decision‐making and subsequent decision regret (DR), and quantify the impact of health‐related quality of life (HRQL) outcomes on DR.

**Methods:**

Men living in the United Kingdom, 18 to 42 months after diagnosis of PCa, were identified from cancer registration data and sent a questionnaire. Measures included the Decision Regret Scale (DRS), Expanded Prostate cancer Index Composite short form (EPIC‐26), EQ‐5D‐5L, and an item on involvement in treatment decision‐making. Multivariable ordinal regression was utilized, with DR categorized as none, mild, or moderate/severe regret.

**Results:**

A total of 17 193 men with stage I‐III PCa completed the DRS: 36.6% reported no regret, 43.3% mild regret, and 20.0% moderate/severe regret. The odds of reporting DR were greater if men indicated their views were not taken into account odds ratio ([OR] = 6.42, 95% CI: 5.39‐7.64) or were involved “to some extent” in decision‐making (OR = 4.63, 95% CI: 4.27‐5.02), compared with men who were “definitely” involved. After adjustment, including for involvement, men reporting moderate/big problems with urinary, bowel, or sexual function were more likely to experience regret compared with men with no/small problems. Better HRQL scores were associated with lower levels of DR.

**Conclusions:**

This large‐scale study demonstrates the benefit of patient involvement in treatment decision‐making for nonmetastatic PCa. However, men experiencing side effects and poorer HRQL report greater DR. Promoting engagement in clinical decision‐making represents good practice and may reduce the risk of subsequent regret.

## 1 BACKGROUND

Clinical decisions taken by patients and clinicians in choosing treatment strategies for localized and locally advanced prostate cancer (PCa) are complex.^1^, ^2^ The range of clinical options that may be appropriate for an individual is diverse, including active surveillance, radical radiotherapy, radical surgery, or androgen deprivation therapy (ADT).^3^ Where treatment options provide comparable levels of long‐term survival,^3^ the significant differences in toxicity^4^, ^5^ and their impact on the quality of survival need to be weighed up carefully in the decision‐making process, yet this is rarely straightforward.

Regret requires imagining possibilities other than the current state being experienced, where individuals reflect on choices and outcomes generated along with considering potential outcomes had the choice been different.^6^ Previous studies exploring decision regret in PCa survivors have been relatively small with the exception of a study by Hoffman et al which included 900 patients.^7^ Recent systematic reviews^5^, ^7^, ^8^ of the decision regret literature suggest studies are limited by the timing of the evaluations carried out, are focused on localized disease, and are limited to comparisons of one or two treatment types. Some have assessed regret immediately after treatment when side effects are unlikely to have developed, while others have evaluated it many years after treatment was completed, when impaired recall might became an issue.

Reviews suggest treatment side effects, such as sexual and urinary dysfunction, and the overall level of patient well‐being are all associated with decision regret.^4^, ^8^ Greater regret has been reported more frequently in patients receiving radical prostatectomy than radical radiotherapy.^4^, ^5^ Patient perception of having made an informed choice (response to the question “I had all the information I needed when a treatment was chosen for my prostate cancer”) was also shown to be significantly associated with regret when patients were evaluated 15 years after initial decision‐making.^7^ For diseases other than PCa, key risk factors for decision regret have included the nature of the decision‐making process, sociodemographic and treatment‐related variables, and poor mental health.^5^, ^9^, ^10^

As part of the Life After Prostate Cancer Diagnosis (LAPCD) study,^11^ a UK‐wide evaluation of quality of life outcomes in over 35 000 PCa survivors, we have collected information on the treatments received, perceived involvement in treatment decision‐making, health‐related quality of life (HRQL) outcomes, and decision regret. With a focus on localized or locally advanced PCa, where men should have some degree of choice over their treatment, we aimed to:

1. assess the relationship between involvement in treatment decision‐making and subsequent decision regret, in order to inform best clinical practice at the time of initial treatment decision‐making;
2. quantify the impact of treatment side effects (urinary, bowel, and sexual function) and HRQL on decision regret.

## 2 METHODS

Men between 18 and 42 months after diagnosis of PCa were identified through cancer registration systems in England, Wales, and Northern Ireland (NI) and through hospital activity data in Scotland. A cross‐sectional postal survey was mailed along with two reminders. In England, 111 of 136 National Health Service (NHS) Trusts participated; 21 declined and 4 were involved in overlapping studies. All providers in NI (n = 5), Scotland (n = 14), and Wales (n = 6) participated. The study protocol is available at https://bmjopen.bmj.com/content/6/12/e013555. A copy of the survey is included as Supplementary File 1. The LAPCD study design has been detailed previously.^11^

The study received approval (under section 251 of the NHS Act) to contact men without prior informed consent. Along with the survey, all men received a detailed patient information sheet. Men consented to take part in the study by returning completed surveys and declined by not returning them, returning them unanswered, or opting out via a free‐phone helpline.

Respondents self‐reported age, ethnicity (categorized as white or nonwhite), marital status (categorized as married/civil partnership, separated/divorced, widowed, single, other), and presence of long‐term conditions (LTCs) from a list of comorbidities (counted and categorized as none, 1, 2, 3, and ≥4). Treatments were self‐reported from a list of possible options and categorized into single therapies (eg, external beam radiotherapy [EBRT] alone) or combinations therapies (eg, EBRT and ADT). Active surveillance and watchful waiting were combined into a “monitoring” category as we believe there was some confusion in understanding the difference between the terms.^12^, ^13^ Stage at diagnosis was obtained from the cancer registration records. Stages I and II were combined into a “localized disease” group and stage III were defined “locally advanced.” A measure of socioeconomic deprivation was derived using area of residence.^14^, ^15^, ^16^, ^17^

Respondents reported their perceived level of involvement in their treatment decision‐making process. This item was taken from the 2014 National Cancer Patient Experience Survey^18^: “Do you think your views were taken into account when the team of doctors and nurses caring for you were discussing which treatment you should have?” This item had five response categories (Yes, definitely; Yes, to some extent; No, my views were not taken into account; I didn't know my treatment was being discussed; Not sure/can't remember).

The Expanded Prostate cancer Index Composite short form (EPIC‐26)^19^ measures function across five domains (urinary incontinence, urinary irritation and obstruction, bowel, sexual, and vitality/hormone). Three items relating to the overall level of problems with urinary, bowel, or sexual function were used, with responses categorized as no/small problem and moderate/big problem. The impact of hormone problems was omitted from analysis due to data collection limitations (hormone problems are strongly linked to ADT use but the survey did not ask whether men were on ADT currently/previous to the time of survey).

EQ‐5D‐5L^20^, ^21^ records information on five dimensions plus a rating of self‐assessed health (SAH) based on the statement “We would like to know how good or bad your health is today” (valued 0‐100, where 100 represents best possible health). Mean SAH ratings were calculated.

The Decision Regret Scale (DRS) is a validated measure of decision regret.^22^ The scale includes five items (It was the right decision; I regret the choice that was made; I would go for the same choice if I had to do it over again; The choice did me a lot of harm; The decision was a wise one) each rated on a five‐point scale. Scores range from 0 to 100 and are calculated in increments of 5. While there are no defined cutoff points for the scale, a recent paper suggested the following cutoff points: No regret 0, Mild regret 5 to 25, and Moderate/severe regret ≥30.^23^

### 2.1 Statistical analysis

Descriptive statistics (chi‐square tests) were used to compare the characteristics of men who did and did not complete the DRS, and to compare the men who reported different levels of regret (no regret, mild, and moderate/severe^23^). Multivariable ordinal regression was performed, with the three categories of decision regret as the outcome variable, which allowed the comparison of mild/moderate/severe regret vs no regret and moderate/severe regret vs mild/no regret. Ordinal regression assumes there is a natural order to the outcome (regret) but the distances between the levels are unknown. Directed acyclic graphs (DAGs) were used to identify appropriate adjustment variables for each model. First, the association between involvement in the decision‐making process and subsequent regret was assessed. This model was adjusted for age, ethnicity, marital status, number of other LTCs, socioeconomic deprivation quintile, and stage of disease as informed by the DAG (Figure S2). Second, associations between HRQL and decision regret were assessed using separate models to look at urinary function, bowel function, sexual function, and self‐assessed health. A combined model looked at the reporting of any functional problem (urinary, and/or bowel, and/or sexual) as men may experience more than one treatment side effect. All HRQL models were adjusted for age, marital status, number of other LTCs, socioeconomic deprivation quintile, treatment type and level of involvement in treatment decision‐making (Figure S4). Data were analyzed using Stata version 15 (StataCorp, Texas).

## 3 RESULTS

### 3.1 Study population

In total, 35 823 men completed a survey (response rate 60.8%, responder and nonresponder characteristics reported in Table S1). The full sample has been described elsewhere.^24^ For the purposes of this study, we focused on men with stage I‐III PCa who reported receiving one of the eight most common treatments (Table S2). Men were excluded if they were diagnosed with stage IV disease (n = 3925), had no stage recorded by the national cancer registration system (n = 5090), reported receiving systemic therapy (n = 320), were unsure about the type of treatment they received (n = 2580) or reported a nonstandard or rare treatment combination (n = 1362). This left a total of 22 358 men, of whom 17 193 (76.9%) answered all five items on the DRS. The level of missing data was low for DRS item 1 (3.8%) but was higher (14.7‐21.2%) for the other four items (Table S3); 12.5% of men answered the first DRS item only. Men were less likely to complete the DRS if they were older, nonwhite, widowed, lived in a more deprived area or were treated with EBRT alone or ADT alone (Table S4).

Table S2 shows the characteristics of respondents to the DRS. The median age at time of survey was 70 years (Interquartile range IQR: 65‐75). Three quarters (74.1%, n = 12 748) were diagnosed with localized (stage I/II) disease and 25.9% (n = 4445) had stage III cancer. The most commonly reported treatments were combined EBRT & ADT (28.1%, n = 4839), surgery alone (28.1%, n = 4824), and monitoring (17.6%, n = 3028).

### 3.2 Decision regret

Just over a third of men (36.6%, n = 6297) reported they had no decision regret, 43.3% (n = 7450) reported mild regret, and 20.0% (n = 3446) reported moderate/severe regret. The characteristics of men reporting different levels of regret are reported in Table S2. Older, nonwhite, separated/divorced or single men, those with other LTCs, and those living in more deprived areas reported higher levels of decision regret. Men who reported receiving ADT alone or in combination with other treatments had higher levels of regret.

### 3.3 Involvement in treatment decision‐making and subsequent decision regret

**Figure 1:**
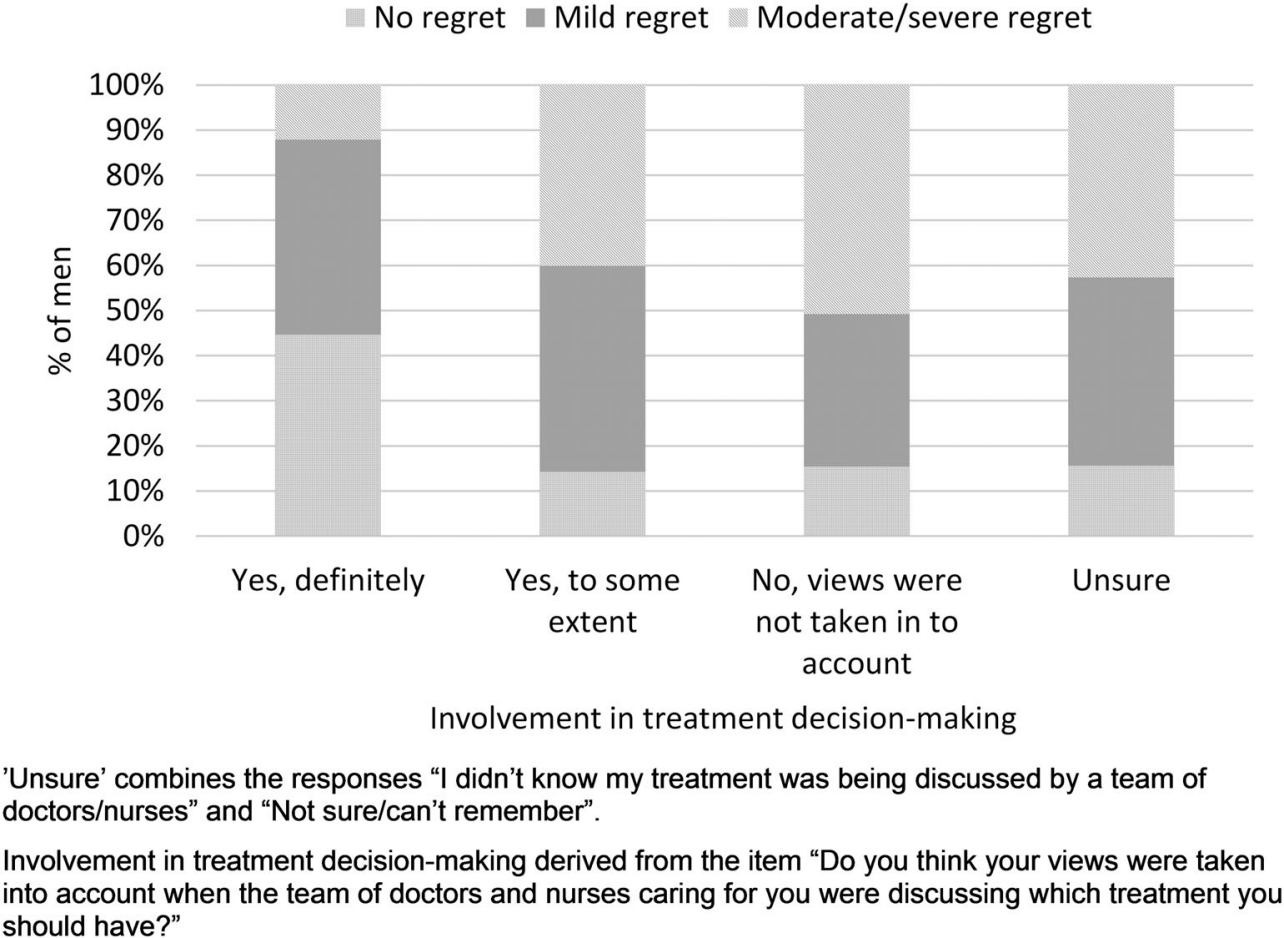
Involvement in treatment decision‐making and experience of decision regret

The majority of men felt their views had “yes, definitely” been taken into account in treatment decision‐making (73.9%; n = 12 574). A further 18.1% (n = 3077) reported they had been involved “yes, to some extent”, while 3.2% (n = 545) reported their views had not been taken into account, and 4.9% (n = 829) were unsure about their involvement in decision‐making (didn't know treatment was being discussed/not sure/can't remember). Men who said they were definitely involved in their treatment decision‐making reported lower levels of regret: 44.6% reported no decision regret, 43.3% reported low regret, and 12.1% reported moderate or high regret (Figure 1). Men who said they were involved “to some extent” reported higher levels of decision regret (no regret: 14.3%; low regret: 45.5%; and moderate/high regret: 40.1%), as did those who said their views were not taken into account (no regret: 15.4%; low regret: 33.8%; and moderate/high regret: 50.8%) (Figure 1).

After adjustment for factors known at the time of treatment decision‐making (including age, ethnicity, number of other LTCs, socioeconomic deprivation quintile, and stage of disease), the odds of reporting decision regret were greater if men indicated “no, my views were not taken into account” (OR = 6.42; 95% CI: 5.39‐7.64), or they were involved “yes, to some extent” (OR = 4.63; 95% CI: 4.27‐5.02) compared with men who reported they were “yes, definitely” involved in decision‐making (Table S5).

### 3.4 The association between quality of life outcomes and decision regret

Overall, 43.9% of men reported that their sexual function was a moderate/big problem. The corresponding figures for urinary and bowel function were 12.0% and 7.9%. Men who reported more severe problems with function experienced higher levels of decision regret (Table 1). Men with greater decision regret had lower mean SAH scores.

**Table 1:** Functional outcomes and level of decision regret

	Level of decision regret			
	None	Mild	Moderate/severe	Total
Moderate/big urinary problem^a^ (n = 17 068)				
No	5844	6560	2621	15 025
	38.9%	43.7%	17.4%	100%
Yes	418	840	785	2043
	20.5%	41.1%	38.4%	100%
Moderate/big bowel problem^a^ (n = 17 089)				
No	6011	6876	2851	15 738
	38.2%	43.7%	18.1%	100%
Yes	244	543	564	1351
	18.1%	40.2%	41.8%	100%
Moderate/big sexual problem^a^ (n = 16 307)				
No	3929	3906	1323	9158
	42.9%	42.7%	14.5%	100%
Yes	2035	3168	1946	7149
	28.5%	44.3%	27.2%	100%
Any moderate/big functional problem^b^ (n = 16 171)				
No	3651	3445	1009	8105
	45.0%	42.5%	12.4%	100%
Yes	2272	3577	2217	8066
	28.2%	44.3%	27.5%	100%
Self‐assessed health^c^ (n = 16 938)				
Mean rating	82.7	78.0	71.3	78.4

a Derived from EPIC‐26 items “Overall, how big a problem has your urinary function/bowel habits/sexual function been for you during the last 4 weeks?”

b Composite variable created including men who reported moderate/severe problems with urinary, bowel, or sexual function.

c Derived from EQ‐5D Visual Analogue Scale “We would like to know how good or bad your health is today” (range 0‐100, where 100 represents the best health you can imagine).

Men may report problems in multiple functional domains and therefore a composite variable was created which included men who reported moderate/severe problems with urinary, bowel, or sexual function. Of the 16 171 men who answered all three items, 49.9% reported one or more moderate/big functional problems. Men with any moderate/big functional problems reported higher levels of decision regret (Table 1).

In the men who said they were “definitely” involved in treatment decision‐making, 53.0% (n = 6292) reported no or small problems with urinary, bowel, or sexual function and 47.0% (n = 5592) reported one or more moderate/big problem. Figure S1 shows the level of decision regret in these groups. Moderate/severe regret was experienced by 7.7% (n = 484) of the no/small functional problems group and 17.1% (n = 957) of the moderate/big functional problems group.

In the men who said their views were not taken into account or they were involved “to some extent” in treatment decision‐making, 42.3% (n = 1749) reported no or small urinary, bowel, or sexual problems and 57.7% (n = 2390) reported one or more moderate/big problem. Moderate/severe regret was experienced by 29.2% (n = 511) of the no/small functional problems group and 51.1% (n = 1221) of the moderate/big functional problems group (Figure S1).

After adjustment for relevant patient characteristics, treatment and involvement in decision‐making, poorer quality of life outcomes were associated with greater decision regret (Table 2). For example, reporting moderate/big problems with bowel function was associated with 2.5 times higher odds of experiencing regret (OR = 2.49, 95% CI: 2.22‐2.79) compared with those reporting no or small problems. The ORs for decision regret in men reporting moderate/big problems with urinary function and sexual function were 2.31 (95% CI: 2.10‐2.54) and 1.93 (95% CI: 1.81‐2.05), respectively, compared with those reporting no or small problems. In men reporting problems with urinary, bowel, or sexual function, the OR of experiencing decision regret was 2.13 (95% CI: 2.00‐2.26). Higher SAH scores were associated with a reduction in the odds of reporting decision regret (OR = 0.98, 95% CI: 0.98‐0.98 per point increase in SAH).

**Table 2:** Multivariable ordinal regression analyses of the association between health‐related quality of life factors and decision regret

Functional outcome	n	%	Adjusted OR^a^	95% CI
Moderate/big urinary problem^b^				
No	15 025	88.0	1.00	
Yes	2043	12.0	2.31	2.10‐2.54
Moderate/big bowel problem^b^				
No	15 738	92.1	1.00	
Yes	1351	7.9	2.49	2.22‐2.79
Moderate/big sexual problem^b^				
No	9158	56.2	1.00	
Yes	7149	43.8	1.93	1.81‐2.05
Any moderate/big functional problem				
No	8105	50.1	1.00	
Yes	8066	49.9	2.13	2.00‐2.26
Self‐assessed health^c^		(Mean)		
Per unit increase in score	16 938	78.4	0.98	0.98‐0.98

Abbreviations: CI, Confidence Interval; OR, Odds ratio.

a The ordinal regression results compare mild/moderate/severe regret vs no regret and moderate/severe regret vs mild/no regret). Models adjusted for age, marital status, number of other long‐term conditions, socioeconomic deprivation quintile, treatment type, and level of involvement in treatment decision‐making.

b Derived from EPIC‐26 items “Overall, how big a problem has your urinary function/bowel habits/sexual function been for you during the last 4 weeks?”

c Derived from the EQ‐5D Visual Analogue Scale “We would like to know how good or bad your health is today” (range 0‐100, where 100 represents the best health you can imagine). This is a continuous variable with results presented as the odds of experiencing decision regret for each unit increase in the self‐assessed health score.

## 4 DISCUSSION

To the best of our knowledge, this is the largest study of decision regret in PCa survivors to date. It includes just under 6000 men with locally advanced disease, a group typically excluded from studies of decision regret, yet who can expect to live for long periods following diagnosis and may be offered treatment choices. Overall, 63% of men reported some level of decision regret regarding their treatment choice. There was a strong association between a perceived lack of involvement in the treatment decision‐making process and greater levels of subsequent decision regret. Men who experienced treatment side effects (poor urinary, bowel, or sexual function) and lower self‐assessed HRQL also reported greater regret about their treatment decision, although the levels of regret were lower in the men who felt they were involved in the decision‐making. Initiatives to promote and support active patient engagement in clinical decision‐making represent good practice and may serve to reduce the risk of subsequent decision regret.

While previous findings from smaller studies have been mixed regarding the association between patient involvement and regret,^25^, ^26^ this study found the odds of experiencing regret were significantly greater if men indicated their views had not been fully taken into account. This is in keeping with an earlier finding from a smaller sample at a much later time point (15 years) from treatment.^7^ In the LAPCD study, approximately one in five men reported moderate or severe regret which is higher than in previous studies.^8^, ^25^, ^27^ One potential explanation for this is the inclusion of men with locally advanced disease as well as the full range of treatment types, whereas previous studies have typically focused on one or two treatment types and those with localized disease. In particular, the inclusion of men who have received ADT, which has been associated with poorer functional^24^ and psychological outcomes^28^ may have increased the magnitude of decision regret being reported.

The strengths of this study lie in its large size and inclusion of the whole population, unbiased by clinical trial selection and not limited by treatment type. Men were between 18 and 42 months after diagnosis, close enough to diagnosis to enable accurate recall but with sufficient time for the potential adverse functional outcomes of treatment to have developed and stabilized.

### 4.1 Study limitations

All measures were completed at the same time and may be subject to mood‐related effects on that specific day. In particular, recall bias may have influenced the responses given, where survivors prior and/or present experiences of side effects may have altered retrospective recall of the decision‐making process.^29^ Just over 60% of men responded to the survey and a quarter of these men did not fully complete the regret scale, hence there may be systematic differences between responders and nonresponders. Men with the poorest outcomes may have been unwilling or unable to complete the survey. Additionally, there were high levels of missing data with up to 20% missing on some items of the DRS. Previous studies using the DRS in PCa have typically questioned individuals in a clinical setting and therefore had little or no missing data. Only men who answered all five components of the DRS were included in this analysis. To our knowledge, this is the largest study to date to use the DRS in a cross‐sectional survey and therefore demonstrates some of the limitations of using this measure.

It may be the case that some men did not feel they had an active “choice” over treatment due to the stage of their disease or other clinical factors. Importantly, men were not asked how involved they wanted to be in their treatment decision‐making. In‐depth qualitative work has demonstrated that not all men were confident or happy in making decisions about their treatment.^30^ For those men with locally advanced disease (stage III) we do not know if they were offered a treatment choice and so there may be conflation between “treatment decision regret” and “treatment regret.”

Due to limitations with cancer registry treatment data, this was self‐reported and therefore relies on accurate recall. A small proportion of men indicated that they had received ADT only. This may be appropriate in some cases,^31^ but it may also be that men did not report all their received treatments. Cancer registry data do not currently include information on disease progression or recurrence; therefore, we did not have up‐to‐date information on disease stage at the time of survey completion. Related to this, we do not know if men had additional treatment at a later date because of disease progression or nonresponse to initial treatment. A lack of detailed information on ADT use (eg, timing and duration) meant we were unable to evaluate the impact of hormone function on decision regret. Finally, we were not able to obtain data on time since diagnosis, which may have influenced men's experience of regret.

### 4.2 Clinical implications

This study reinforces the need for clinicians to fully explain the likely consequences of different treatment options, including specific functional problems, during up‐front decision‐making consultations. Men with PCa, and partners or family members where possible and appropriate, should be actively involved in discussions about their treatment. Clinicians caring for men with PCa should be reassured that patient involvement in decision‐making is not only intuitively representative of best practice but may also enhance quality of survival through reduced subsequent decision regret.

## 5 CONCLUSIONS

This large‐scale study provides evidence of the benefit of patient involvement in treatment decision‐making for localized and locally advanced PCa. However, due to the nature of the treatments involved, there will always be a group of men who experience treatment‐related late effects, and appropriate support should be available to reduce the impact of these late effects on their wider quality of life.

## CONFLICT OF INTEREST

A.W.G. reports grants from Candlelighters, grants from Macmillan Cancer Support, grants from NIHR, grants from Yorkshire Cancer Research, outside the submitted work. All other authors declare no conflict of interests.

## AUTHOR CONTRIBUTIONS

Conception and design: A.G. and A.W.G. are co‐Principal Investigators and designed the study together with co‐investigators A.D., P.W., L.H., P.S., E.W., R.W., P.K., and H.B.

Collection and assembly of data: R.M., M.A., and T.K.

Data analysis and interpretation: S.W., A.D., A.W.G., D.D., W.C., P.S., P.W., E.W., and R.W.

Manuscript writing: All authors.

Final approval of manuscript: All authors.

Accountable for all aspects of the work: All authors.

## ETHICS STATEMENT

The study received the following approvals: Newcastle & North Tyneside Research Ethics Committee (15/NE/0036), Confidentiality Advisory Group (15/CAG/0110), NHS Scotland Public Benefit and Privacy Panel (0516‐0364) and NHS R&D approval from Wales, Scotland, and Northern Ireland.

## Supporting information


**Figure S1** Level of decision regret split by functional outcome and perceived involvement in treatment decision‐making

**Figure S2** Directed acyclic graph assessing potential confounders of the relationship between involvement in treatment decision‐making and decision regret

**Figure S3** Directed acyclic graph assessing potential confounders of the relationship between quality of life outcomes and decision regret

**Figure S4** Study inclusions, exclusions and response rates

**Supplementary File 1** Copy of the LAPCD survey
Supplementary File 2. Treatment data additional details
Supplementary Table 1: Comparison of responders and nonresponders
Supplementary Table 2. Sociodemographic and clinical characteristics by level of decision regret
Supplementary Table 3. Completeness of each item within the decision regret scale
Supplementary Table 4. Characteristics of nonresponders to the decision regret scale
Supplementary Table 5. Multivariable ordinal regression analysis of the association between involvement in decision‐making and decision regret
Supplementary Table 6. STROBE statement: checklist for cross sectional studies

## Data Availability

The datasets generated and/or analyzed in the current study are not available publicly as eligible patients were informed at the time of the survey their data would be stored securely and confidentially. The processes for accessing the data used are available from the corresponding author.

## References

[1] Jani AB , Hellman S . Early prostate cancer: clinical decision‐making. Lancet. 2003;361(9362):1045‐1053. 12660074 10.1016/S0140-6736(03)12833-4

[2] Chen RC , Clark JA , Manola J , Talcott JA . Treatment 'mismatch’ in early prostate cancer: do treatment choices take patient quality of life into account? Cancer. 2008;112(1):61‐68. 18040996 10.1002/cncr.23138

[3] Hamdy FC , Donovan JL , Lane JA , et al. 10‐year outcomes after monitoring, surgery, or radiotherapy for localized prostate cancer. N Engl J Med. 2016;375(15):1415‐1424. 27626136 10.1056/NEJMoa1606220

[4] Christie DR , Sharpley CF , Bitsika V . Why do patients regret their prostate cancer treatment? A systematic review of regret after treatment for localized prostate cancer. Psychooncology. 2015;24(9):1002‐1011. 25728586 10.1002/pon.3776

[5] Diefenbach MA , Mohamed NE . Regret of treatment decision and its association with disease‐specific quality of life following prostate cancer treatment. Cancer Invest. 2007;25(6):449‐457. 17882657 10.1080/07357900701359460

[6] Pieters R , Zeelenberg M . A theory of regret regulation 1.1. J Consum Psychol. 2007;17(1):29‐35. 10.1207/s15327663jcp1701_5 PMC2435165 18568095

[7] Hoffman RM , Lo M , Clark JA , et al. Treatment decision regret among long‐term survivors of localized prostate cancer: results from the prostate cancer outcomes study. J Clin Oncol. 2017;35(20):2306‐2314. 28493812 10.1200/JCO.2016.70.6317 PMC5501361

[8] Becerra Perez MM , Menear M , Brehaut JC , Legare F . Extent and predictors of decision regret about health care decisions: a systematic review. Med Decis Making. 2016;36(6):777‐790. 26975351 10.1177/0272989X16636113

[9] Hurwitz LM , Cullen J , Kim DJ , et al. Longitudinal regret after treatment for low‐and intermediate‐risk prostate cancer. Cancer. 2017;123(21):4252‐4258. 28678408 10.1002/cncr.30841

[10] Maguire R , Hanly P , Drummond FJ , Gavin A , Sharp L . Regret and fear in prostate cancer: the relationship between treatment appraisals and fear of recurrence in prostate cancer survivors. Psychooncology. 2017;26(11):1825‐1831. 28124398 10.1002/pon.4384

[11] Downing A , Wright P , Wagland R , et al. Life after prostate cancer diagnosis: protocol for a UK‐wide patient‐reported outcomes study. BMJ Open. 2016;6(12). 10.1136/bmjopen-2016-013555 PMC5168696 27927667

[12] Mallapareddi A , Ruterbusch J , Reamer E , Eggly S , Xu J . Active surveillance for low‐risk localized prostate cancer: what do men and their partners think? Fam Pract. 2017;34(1):90‐97. 28034917 10.1093/fampra/cmw123 PMC6916739

[13] Kim C , Wright FC , Look Hong NJ , et al. Patient and provider experiences with active surveillance: a scoping review. PLOS One. 2018;13(2):e0192097. 29401514 10.1371/journal.pone.0192097 PMC5798833

[14] Department for Communities and Local Government . English Indices of Multiple Deprivation 2010. https://www.gov.uk/government/publications/english-indices-of-deprivation-2010. Published March 24, 2011. Accessed March 9, 2013.

[15] ISD Scotland . The Scottish Index of Multiple Deprivation (SIMD). 2017; http://www.isdscotland.org/Products-and-Services/GPD-Support/Deprivation/SIMD/.

[16] Northern Ireland Statistics and Research Agency . NI Multiple Deprivation Measure. 2010; https://www.nisra.gov.uk/statistics/deprivation/northern-ireland-multiple-deprivation-measure-2010-nimdm2010. Accessed March 10, 2017.

[17] Welsh Government . Welsh Index of Multiple Deprivation. 2017; http://wimd.wales.gov.uk/?lang=en. Accessed October 4, 2017.

[18] Quality Health . National Cancer Patient Experience Survey. 2015; http://www.quality-health.co.uk/surveys/national-cancer-patient-experience-survey. Accessed March 3, 2015, 2015.

[19] Wei JT , Dunn RL , Litwin MS , Sandler HM , Sanda MG . Development and validation of the expanded prostate cancer index composite (EPIC) for comprehensive assessment of health‐related quality of life in men with prostate cancer. Urology. 2000;56(6):899‐905. 11113727 10.1016/s0090-4295(00)00858-x

[20] EuroQol Group . EQ‐5D‐5L value sets. http://www.euroqol.org/about-eq-5d/valuation-of-eq-5d/eq-5d-5l-value-sets.html. Accessed April 9, 2013.

[21] Herdman M , Gudex C , Lloyd A , et al. Development and preliminary testing of the new five‐level version of EQ‐5D (EQ‐5D‐5L). Qual Life Res. 2011;20(10):1727‐1736. 21479777 10.1007/s11136-011-9903-x PMC3220807

[22] Brehaut JC , O'Connor AM , Wood TJ , et al. Validation of a decision regret scale. Med Decis Making. 2003;23(4):281‐292. 12926578 10.1177/0272989X03256005

[23] Becerra‐Perez M‐M , Menear M , Turcotte S , Labrecque M , & Légaré F . (2016). More primary care patients regret health decisions if they experienced decisional conflict in the consultation: a secondary analysis of a multicenter descriptive study. BMC Family Practice, 17(1). 10.1186/s12875-016-0558-0 PMC5103443 27832752

[24] Downing A , Wright P , Hounsome L , et al. Quality of life in men living with advanced and localised prostate cancer in the UK: a population‐based study. Lancet Oncol. 2019;20(3):436‐447. 30713036 10.1016/S1470-2045(18)30780-0

[25] Davison BJ , So AI , Goldenberg SL . Quality of life, sexual function and decisional regret at 1 year after surgical treatment for localized prostate cancer. BJU Int. 2007;100(4):780‐785. 17578466 10.1111/j.1464-410X.2007.07043.x

[26] Soeyonggo T , Warde P , Fleshner N , Timilshina N , Alibhai SM . Information needs of men on androgen deprivation therapy. BJU Int. 2012;109(10):1503‐1509. 21883845 10.1111/j.1464-410X.2011.10475.x

[27] Hu JC , Kwan L , Krupski TL , et al. Determinants of treatment regret in low‐income, uninsured men with prostate cancer. Urology. 2008;72(6):1274‐1279. 18313115 10.1016/j.urology.2007.11.066

[28] Cary KC , Singla N , Cowan JE , Carroll PR , Cooperberg MR . Impact of androgen deprivation therapy on mental and emotional well‐being in men with prostate cancer: analysis from the CaPSURE registry. J Urol. 2014;191(4):964‐970. 24184370 10.1016/j.juro.2013.10.098

[29] Schmier JK , Halpern MT . Patient recall and recall bias of health state and health status. Expert Rev Pharmacoecon Outcomes Res. 2004;4(2):159‐163. 19807519 10.1586/14737167.4.2.159

[30] Wagland R , Nayoan J , Matheson L , et al. Very difficult for an ordinary guy’: factors influencing the quality of treatment decision‐making amongst men diagnosed with localised and locally advanced prostate cancer: findings from a UK‐wide mixed methods study. Patient Educ Couns. 2019;102(4):797‐803. 30527732 10.1016/j.pec.2018.12.004

[31] Mottet N , van den Bergh RCN , Briers E , Bourke L , Cornford P , De Santis M , Gillessen S , Govorov A , Grummet J , Henry AM , Lam TB , Mason MD , van der Poel HG , van der Kwast TH , Rouvière O , Wiegel T , Van den Broeck T , Cumberbatch M , Fossati N , Gross T , Lardas M , Liew M , Moris L , Schoots IG , Willemse PM et al. EAU‐ESTRO‐SIOG guidelines on prostate cancer 2018 https://uroweb.org/wp‐content/uploads/EAU‐ESUR‐ESTRO‐SIOG‐Guidelines‐on‐Prostate‐Cancer‐large‐text‐V2.pdf. Accessed January 13, 2020.

