# Supplementary figures and images for: Decision regret in men living with and beyond nonmetastatic prostate cancer in the United Kingdom: A population‐based patient‐reported outcome study
Source: Psychooncology. 2020 Feb 26;29(5):886–93. doi: 10.1002/pon.5362 (PMC7317932; doi:10.1002/pon.5362)

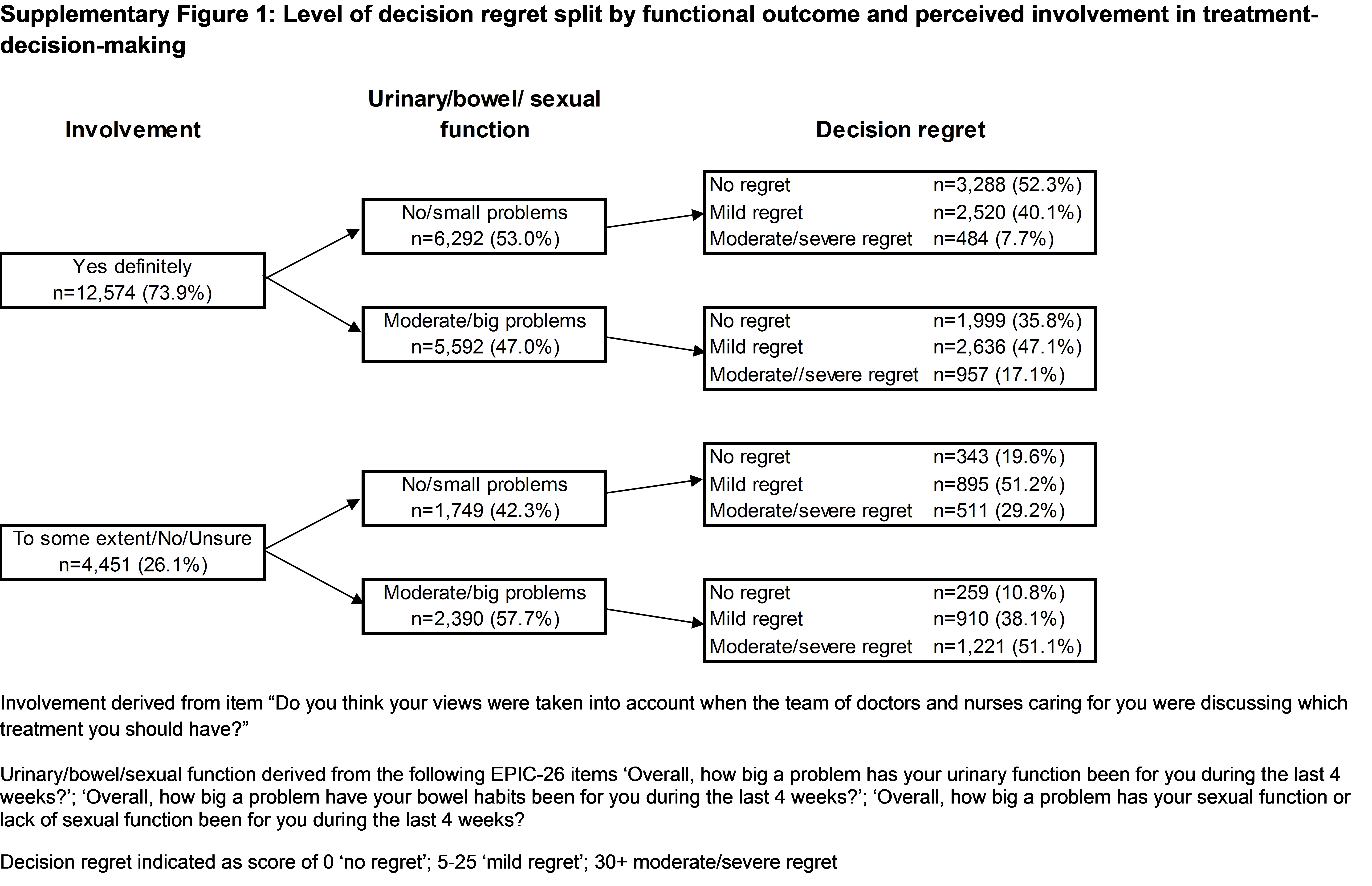

Supplement: Supplementary file 1 — Figure S1 Level of decision regret split by functional outcome and perceived involvement in treatment decision‐making [file PON-29-886-s001.jpg]

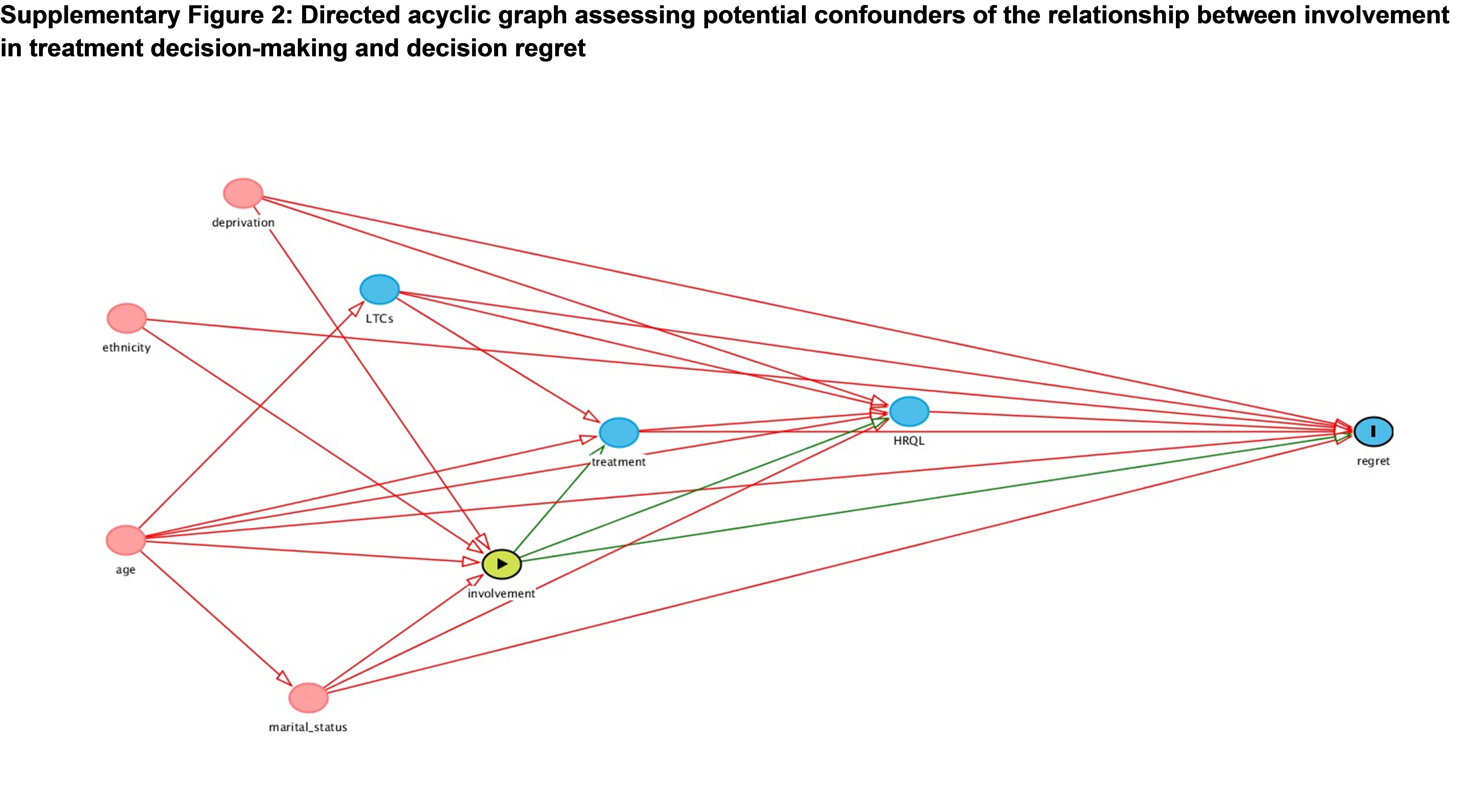

Supplement: Supplementary file 2 — Figure S2 Directed acyclic graph assessing potential confounders of the relationship between involvement in treatment decision‐making and decision regret [file PON-29-886-s002.jpg]

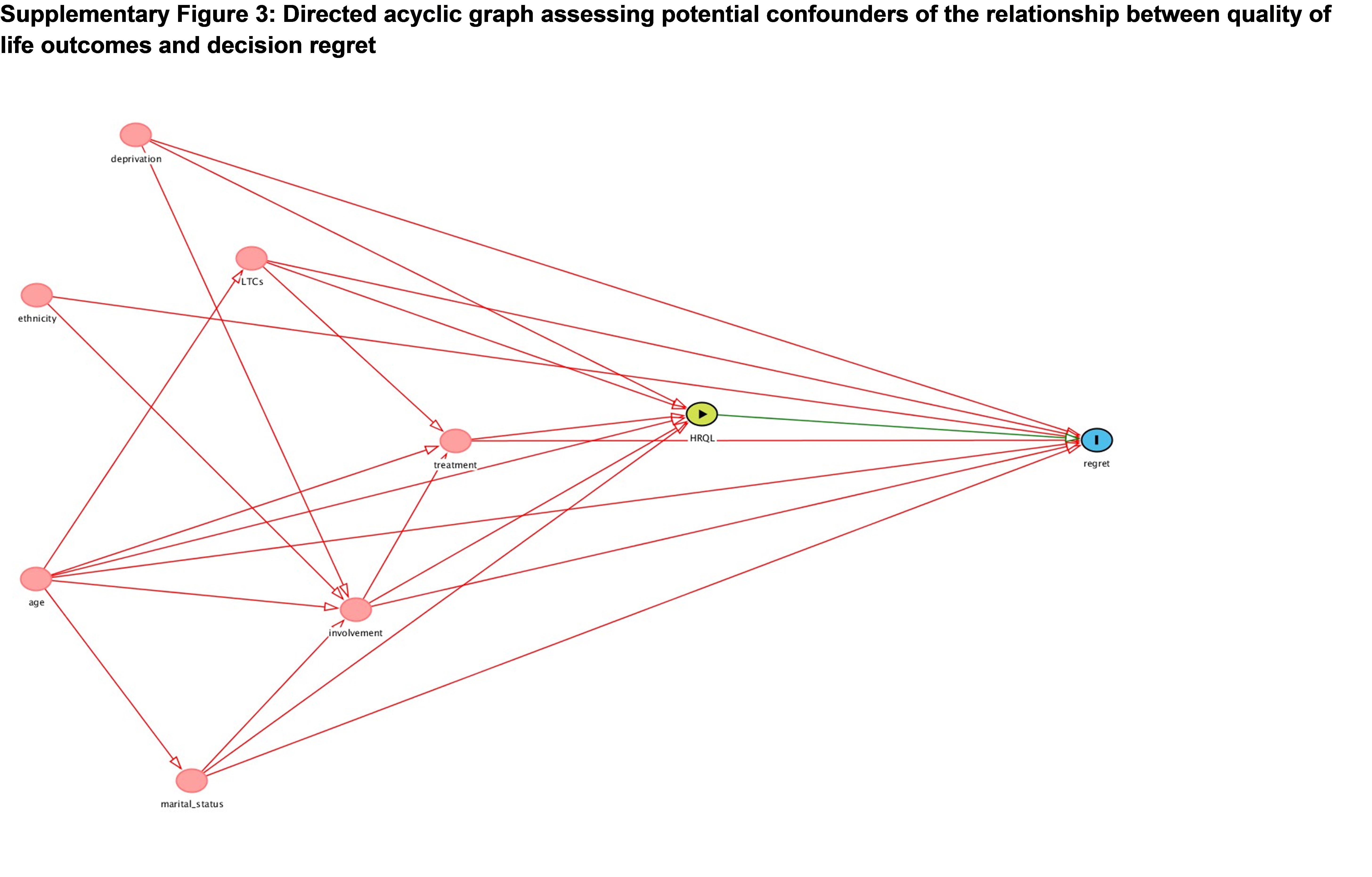

Supplement: Supplementary file 3 — Figure S3 Directed acyclic graph assessing potential confounders of the relationship between quality of life outcomes and decision regret [file PON-29-886-s003.jpg]

**Supplementary Figure 4: Study inclusions, exclusions and response rates**

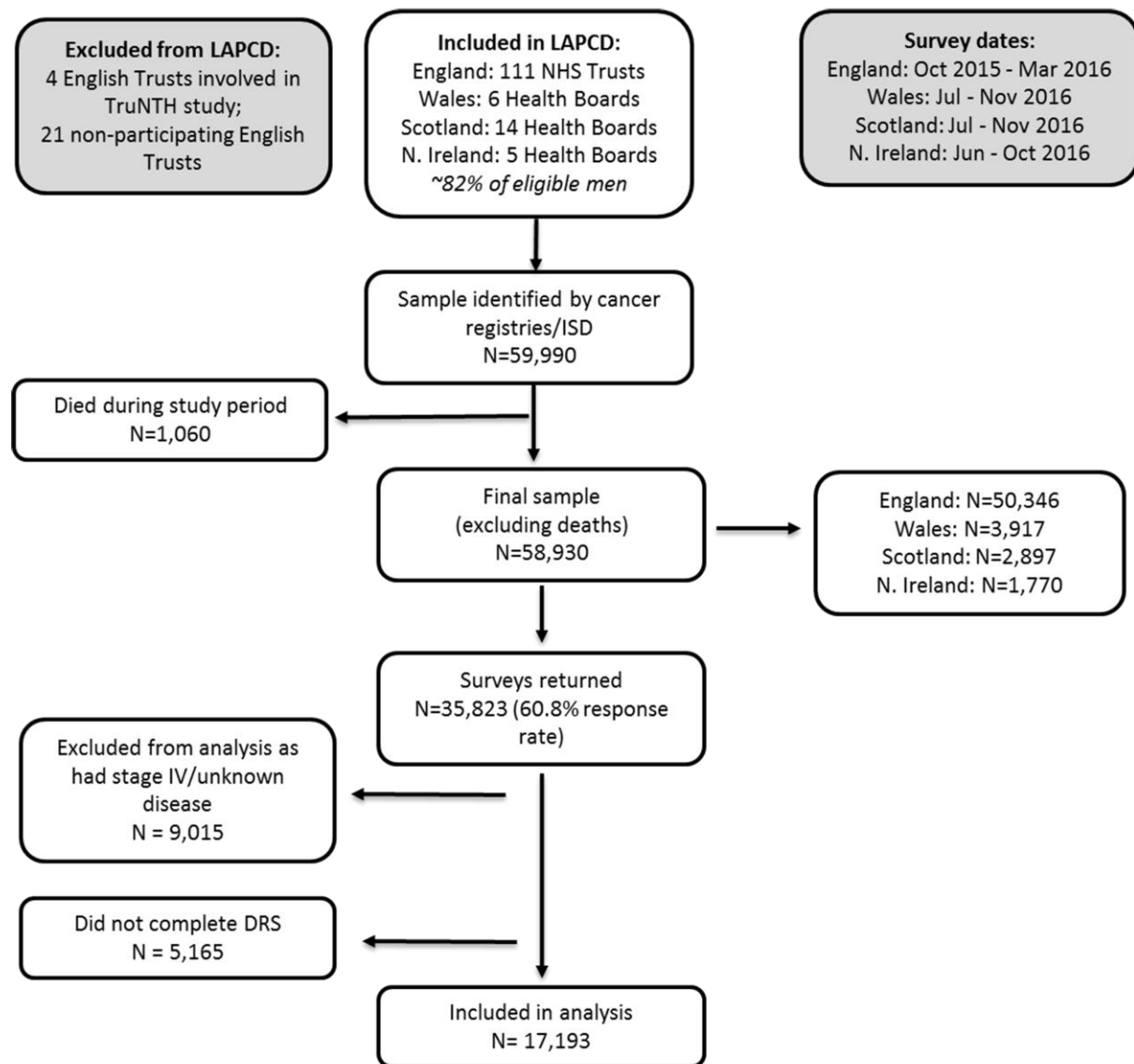

NI: Northern Ireland

Supplement: Supplementary file 4 — Figure S4 Study inclusions, exclusions and response rates [file PON-29-886-s004.pdf]
