# Supplementary material for: Decision regret in men living with and beyond nonmetastatic prostate cancer in the United Kingdom: A population‐based patient‐reported outcome study
Source: Psychooncology. 2020 Feb 26;29(5):886–93. doi: 10.1002/pon.5362 (PMC7317932; doi:10.1002/pon.5362)
Supplement: Supplementary file 5 — Supplementary File 1 Copy of the LAPCD survey Supplementary File 2. Treatment data additional details Supplementary Table 1: Comparison of responders and nonresponders Supplementary Table 2. Sociodemographic and clinical characteristics by level of decision regret Supplementary Table 3. Completeness of each item within the decision regret scale Supplementary Table 4. Characteristics of nonresponders to the decision regret scale Supplementary Table 5. Multivariable ordinal regression analysis of the association between involvement in decision‐making and decision regret Supplementary Table 6. STROBE statement: checklist for cross sectional studies [file PON-29-886-s005.docx]

**Appendices**

**Supplementary File 1: Copy of the LAPCD survey**

**Supplementary File 2. Treatment data additional details**

**Supplementary Table 1: Comparison of responders and non-responders**

**Supplementary Table 2.** **Sociodemographic and clinical characteristics by level of decision regret**

**Supplementary Table 3. Completeness of each item within the decision regret scale**

**Supplementary Table 4. Characteristics of non-responders to the decision regret scale**

**Supplementary Table 5. Multivariable ordinal regression analysis of the association between involvement in decision-making and decision regret**

**Supplementary Figure 1: Level of decision regret split by functional outcome and perceived involvement in treatment-decision-making**

**Supplementary Figure 2: Directed acyclic graph assessing potential confounders of the relationship between involvement in treatment decision-making and decision regret**

**Supplementary Figure 3: Directed acyclic graph assessing potential confounders of the relationship between quality of life outcomes and decision regret**

**Supplementary Figure 4: Study inclusions, exclusions and response rates**

**Supplementary Table 6. STROBE statement: checklist for cross sectional studies**

**Supplementary File 1: Copy of the LAPCD survey**

**Life After Prostate Cancer Diagnosis:**

**Patient Reported Outcomes Survey**

More men are now living longer after a diagnosis of prostate cancer. We want to find out what life is really like for this group of men. Your answers will benefit other men with prostate cancer in the future by providing information to help clinical teams, service providers and policy makers make decisions about how to improve the quality of services for prostate cancer patients. We would be grateful if you would complete this survey, which asks for information about your health and quality of life.

If you have **not** had a diagnosis of prostate cancer this questionnaire is not relevant to you. Please tick the “no” box below and please accept our apologies for contacting you. Please return the blank questionnaire in the envelope provided and we will correct our records. If you have any questions about this survey please contact the FREEPHONE helpline number: **0808 801 0678**.

Reference Number

**Have you ever been told by a doctor that you have prostate cancer?**

**Yes No**

If you have ticked yes to the first question please complete the rest of the survey.

If you have ticked no, please accept our apologies and send the questionnaire back to us in the envelope provided.

**The survey**

This survey is made up of eight sections and will take approximately 30 minutes to complete.

**Who should complete the questionnaire?**

The questions should be answered by the person named in the letter that came with this questionnaire. If that person needs help to answer the questions then the answers should be given from their point of view – not from the point of view of the person who is helping.

**Completing the questionnaire**

For each question please tick clearly inside the box of the response that best represents your views, using a black or blue pen. Do not worry if you make a mistake. Just cross out the mistake and put a tick in the correct box. Do not write your name or address anywhere on the questionnaire. The more questions in this survey that you complete, the more we can understand what life is like for those living with and beyond prostate cancer. However, if you feel unable or uncomfortable about answering any of the questions, leave it blank and move on to the next one.

The information you give us will be kept **securely** and treated in **confidence**. We will not publish any personal information that could allow anyone to identify you. We are very grateful for your time and effort in completing this survey.

If you have any queries about the questionnaire, please call the FREEPHONE helpline number: **0808 801 0678**

You can find more information about the study at: http://www.lifeafterprostatecancerdiagnosis.com/

Section One: Your overall health

Under each heading, please tick ONE box that best describes your health TODAY

**1. MOBILITY**

I have no problems in walking about ❑

I have slight problems in walking about ❑

I have moderate problems in walking about ❑

I have severe problems in walking about ❑

I am unable to walk about ❑

**2. SELF-CARE**

I have no problems washing or dressing myself ❑

I have slight problems washing or dressing myself ❑

I have moderate problems washing or dressing myself ❑

I have severe problems washing or dressing myself ❑

I am unable to wash or dress myself ❑

**3. USUAL ACTIVITIES** (e.g. work, study, housework, family or leisure activities)

I have no problems doing my usual activities ❑

I have slight problems doing my usual activities ❑

I have moderate problems doing my usual activities ❑

I have severe problems doing my usual activities ❑

I am unable to do my usual activities ❑

**4. PAIN / DISCOMFORT**

I have no pain or discomfort ❑

I have slight pain or discomfort ❑

I have moderate pain or discomfort ❑

I have severe pain or discomfort ❑

I have extreme pain or discomfort ❑

**5. ANXIETY / DEPRESSION**

I am not anxious or depressed ❑

I am slightly anxious or depressed ❑

I am moderately anxious or depressed ❑

I am severely anxious or depressed ❑

I am extremely anxious or depressed ❑

**Are there any additional HEALTH issues that are of concern to you?**

*© 1998 EuroQol GroupEQ-5D*™ *is a trade mark of the EuroQol Group*

*© 1998 EuroQol GroupEQ-5D*™ *is a trade mark of the EuroQol Group*

**6. We would like to know how good or bad your health is TODAY.**

- This scale is numbered from 0 to 100.

The best health

you can imagine

The worst health

you can imagine

The worst health

you can imagine

- 100 means the best health you can imagine.

0 means the worst health you can imagine.

- Mark an X on the scale to indicate how your health is TODAY.
- Now, please write the number you mark on the scale in the box below.

YOUR HEALTH TODAY =

Section Two: Your diagnosis and treatment

1. How were you diagnosed? *Please* *tick* ***all that apply***

|  | I attended my GP with urinary symptoms (e.g. urinating frequently, blood in urine) |
| --- | --- |
|  | I attended my GP with other symptoms (e.g. back pain, joint pain) |
|  | I had no symptoms and my GP offered to test my PSA (blood test) as part of a general health check |
|  | I had no symptoms and I asked my GP to measure my PSA (blood test)  I had symptoms and I asked my GP to measure my PSA (blood test) |
|  | I had a PSA test as part of a private health check |
|  | Other |

I attended my GP with other symptoms (e.g. back pain, joint pain)

I had no symptoms and my GP offered to test my PSA (blood test) as part of a general health check

I had no symptoms and I asked my GP to measure my PSA (blood test)

I had a PSA test as part of a private health check

Other

Please tell us about this:

**8.** Please tell us which treatments you have had following your diagnosis of prostate cancer *Please tick* ***all*** *the options that apply.*

|  | | |  | |
| --- | --- | --- | --- | --- |
| **A. Have you had surgery (prostatectomy)?**  If **no**, go to **B**  If **yes**, what type of surgery? Please tick one box | No | Yes | | |
| **Open prostatectomy**  *Operation performed through a cut in the abdomen above the pubic bone area (retropubic prostatectomy) or a cut in the area between the testicles and back passage (perineal prostatectomy).* | | | |  |
| **Laparoscopic (keyhole) prostatectomy**  *Operation performed through small incisions in the abdominal wall.* | | | |  |
| **Robotic prostatectomy**  *Operation performed with the assistance of a surgical robot (Da Vinci prostatectomy).* | | | |  |
| **I don't know what kind of operation I had** | | | |  |
|  | | | |  |
| **B. Have you had radiotherapy?**  If **no**, go to **C**  If **yes**, what type of radiotherapy? Please tick all that apply | No | Yes | | |
| **External beam radiotherapy (with or without hormone treatment)**  *Radiotherapy uses high-energy X-ray beams to treat the whole prostate. This form of treatments includes both 3-dimensional conformal radiotherapy (3D-CRT) and intensity modulated radiotherapy (IMRT).* | | | |  |
| **Permanent seed (low-dose) brachytherapy**  *This involves implanting radioactive seeds into the prostate gland.* | | | |  |
| **Temporary (high-dose) brachytherapy (with or without external beam radiotherapy or hormone treatment)**  *This involves inserting a source of high-dose radiation into the prostate gland for a few minutes.* | | | |  |
| **I don’t know what type of radiotherapy or brachytherapy I had**  **C. Have you had any of the following treatments?**  **Please tick all that apply.** | | | |  |
| \| **High intensity focused ultrasound (HIFU)**  *This treatment uses ultrasound waves to heat and destroy cancer cells in the prostate.* \|  \|  \|  \| \| --- \| --- \| --- \| --- \| \| **Cryotherapy**  *This treatment uses freezing and thawing to kill the cancer cells in the prostate.* \|  \|  \|  \| \| **Chemotherapy** (not including hormones) \|  \|  \|  \| \| **Hormone treatment** (either continuous or on/off treatment) \|  \|  \|  \| \| **Abiraterone and/or Enzalutamide** \|  \|  \|  \| | | | |  |

**D. Are doctors and nurses currently monitoring your prostate cancer?**

| If **no**, go to question **9**  If **yes**, what type of monitoring? Please tick one box. | No | Yes |
| --- | --- | --- |

| ***Active Surveillance***  *Surveillance is monitoring of low risk, slow growing* ***localised prostate cancer*** *with the aim of avoiding or delaying* ***curative treatment*** *(e.g. surgery, radiotherapy).This involves having regular tests.* |  |
| --- | --- |
| ***Watchful waiting***  *Watchful waiting is a way of monitoring prostate cancer that isn’t causing any symptoms or problems. The aim is to keep an eye on the cancer over the long term and only having treatment if the cancer deteriorates or the patient gets symptoms. This involves fewer tests than in active surveillance.* |  |
| ***Clinical follow-up during or after one of the treatments mentioned above in 8A, B or C*** |  |
| ***I am unsure about the type of monitoring I am currently having*** |  |

**9a.** Do you think your views were taken into account when the team of doctors and nurses caring for you were discussing which treatment you should have? Please tick one of the following boxes:

 Yes, definitely

 Yes, to some extent

 No, my views were not taken into account

 I didn’t know my treatment was being discussed by a team of doctors / nurses  Not sure / can’t remember

**9b.** Please answer the following questions whether or not you were actively involved in the decisions made about your treatment.

Please think about the decisions you made about your treatment for prostate cancer after talking to your doctor, surgeon, nurse, health care professional etc.

*Please show how you feel about these statements by ticking* ***one*** *box on each row.*

|  | Strongly agree | Agree | Neither agree nor disagree | Disagree | Strongly disagree |
| --- | --- | --- | --- | --- | --- |
| a. It was the right decision |  |  |  |  |  |
| b. I regret the choice that was made |  |  |  |  |  |
| c. I would go for the same choice if I had to do it over again |  |  |  |  |  |
| d. The choice did me a lot of harm |  |  |  |  |  |
| e. The decision was a wise one |  |  |  |  |  |

**Please add anything else you would like to tell us about your diagnosis, treatment, and the decision making process.**

Section Three: How things are for you now

We understand that some of the following questions are very sensitive, but we would really appreciate you answering them if possible. As with the rest of the questionnaire, your answers will be kept confidential and no one will be able to identify you.

*Please tick* ***one*** *box for each question.*

| 10. | Over the past 4 weeks, how often have you leaked urine? |
| --- | --- |
| More than once a day |  |
| About once a day |  |
| More than once a week |  |
| About once a week |  |
| Rarely or never |  |

| 11. | Which of the following best describes your urinary control during the last 4 weeks? |
| --- | --- |
| No urinary control whatsoever |  |
| Frequent dribbling |  |
| Occasional dribbling |  |
| Total control |  |

| 12. | How many pads per day did you usually use to control leakage during the last 4 weeks? |
| --- | --- |
| None |  |
| 1 pad per day |  |
| 2 pads per day |  |
| 3 or more pads per day |  |

| 13. | How big a problem, if any, has each of the following been for you during the last 4 weeks?  *Please tick one box on each line.* | | | | |
| --- | --- | --- | --- | --- | --- |
|  | No problem | Very small problem | Small problem | Moderate problem | Big problem |
| a. Dripping or leaking urine |  |  |  |  |  |
| b. Pain or burning on urination |  |  |  |  |  |
| c. Bleeding with urination |  |  |  |  |  |
| d. Weak urine stream or incomplete emptying |  |  |  |  |  |
| e. Need to urinate frequently during the day |  |  |  |  |  |

| 14. | Overall, how big a problem has your urinary function been for you during the last 4 weeks?  *Please tick one box.* |
| --- | --- |
| No problem |  |
| Very small problem |  |
| Small problem |  |
| Moderate problem |  |
| Big problem |  |

| 15. | How big a problem, if any, has each of the following been for you during the last 4 weeks?  *Please tick one box on each line.* | | | | |
| --- | --- | --- | --- | --- | --- |
|  | No problem | Very small problem | Small problem | Moderate problem | Big problem |
| a. Urgency to have a bowel movement | 1 |  |  |  |  |
| b. Increased frequency of bowel movements |  |  |  |  |  |
| c. Losing control of your stools |  |  |  |  |  |
| d. Bloody stools |  |  |  |  |  |
| e. Abdominal/ Pelvic/Rectal pain |  |  |  |  |  |

| 16. | Overall, how big a problem have your bowel habits been for you during the last 4 weeks?  *Please tick one box.* |
| --- | --- |
| No problem |  |
| Very small problem |  |
| Small problem |  |
| Moderate problem |  |
| Big problem |  |

| 17. | How would you rate each of the following during the last 4 weeks? *Please tick one box on each line.* | | | | |
| --- | --- | --- | --- | --- | --- |
|  | Very poor to none | Poor | Fair | Good | Very good |
| a. Your ability to have an erection | 1 |  |  |  |  |
| b. Your ability to reach orgasm (climax) |  |  |  |  |  |

| 18. | How would you describe the usual QUALITY of your erections during the last 4 weeks?  *Please tick one box.* |
| --- | --- |
| None at all |  |
| Not firm enough for any sexual activity |  |
| Firm enough for masturbation and foreplay only |  |
| Firm enough for intercourse |  |

*Please tick* ***one*** *box for each question.*

| 19. | How would you describe the FREQUENCY of your erections during the last 4 weeks? |
| --- | --- |
| I NEVER had an erection when I wanted one |  |
| I had an erection LESS THAN HALF the time I wanted one |  |
| I had an erection ABOUT HALF the time I wanted one |  |
| I had an erection MORE THAN HALF the time I wanted one |  |
| I had an erection WHENEVER I wanted one |  |

| 20. | Overall, how would you rate your ability to function sexually during the last 4 weeks? |
| --- | --- |
| Very poor |  |
| Poor |  |
| Fair |  |
| Good |  |
| Very good |  |

| 21. |  | Overall, how big a problem has your sexual function or lack of sexual function been for you during the last 4 weeks? |
| --- | --- | --- |
| No problem |  |  |
| Very small problem |  |  |
| Small problem |  |  |
| Moderate problem |  |  |
| Big problem |  |  |

| 22. | How big a problem during the last 4 weeks, if any, has each of the following been for you?  *Please tick one box on each line.* | | | | |
| --- | --- | --- | --- | --- | --- |
|  | No problem | Very small problem | Small problem | Moderate problem | Big problem |
| Hot flushes | 1 |  |  |  |  |
| Breast tenderness/enlargement |  |  |  |  |  |
| Feeling depressed |  |  |  |  |  |
| Lack of energy |  |  |  |  |  |
| Change in body weight |  |  |  |  |  |

| **During the last 4 weeks** *Please tick* ***one*** *box on each line.* | | | | |
| --- | --- | --- | --- | --- |
|  | Not  at all | A  little | Quite a bit | Very much |
| **23.** To what extent were you interested in sex? |  |  |  |  |
| **24**. To what extent were you sexually active (with or without intercourse)? |  |  |  |  |

| 25. Have you used any medications to aid or improve erections since your prostate cancer diagnosis? (e.g. tablets, penis injections, gels) *Please tick one box.* | | | | | | |
| --- | --- | --- | --- | --- | --- | --- |
|  |  |  |  |  |  |  |
| I was not offered this | I was offered this but did not want it | I was offered this but have not tried it | I was offered this and tried it, but it  was not helpful | I was offered this and it helped,  but I am  not using  it now | I was offered this, it helps  and I use it  sometimes | I was offered this, it helps  and I use it often |

| 26. Have you used any devices to aid or improve erections since your prostate cancer diagnosis? (e.g. vacuum pump, penile prosthesis) *Please tick one box.* | | | | | | | | | | | |
| --- | --- | --- | --- | --- | --- | --- | --- | --- | --- | --- | --- |
|  |  | |  | |  | |  | |  | |  |
| I was not offered this | I was offered this but did not want it | | I was offered this but have not tried it | | I was offered this and tried it, but it  was not helpful | | I was offered this and it helped,  but I am  not using  it now | | I was offered this, it helps  and I use it  sometimes | | I was offered this, it helps  and I use it often |
| 27. Have you used any specialist services to help with your sex life following your diagnosis of prostate cancer? (e.g. counselling, psychosexual clinics, psychology)  *Please tick one box.* | | | | | | | | | | | |
|  | |  | |  | |  | |  | |  | |
| I was not offered this | | I was offered this but did not want it | | I was offered this but have not tried it | | I was offered this and tried it, but it  was not helpful | | I was offered this and it helped,  but I am  not using  it now | | I was offered this, it helps  and I am still using the service | |

| **During the past week:** *Please tick* ***one*** *box on each line.* | | | | | | |
| --- | --- | --- | --- | --- | --- | --- |
|  | Not  at all | A  little | Quite a bit | | Very much | |
| **28.** Did you need to rest? |  |  | |  | |  |
| **29**. Have you felt weak? |  |  | |  | |  |
| **30.** Were you tired? |  |  | |  | |  |

**Please add anything else you would like to tell us about your symptoms or the side effects of your treatment.**

Section Four: Your everyday life

On each line please tick the box that best describes your answer.

Please tick the **‘no difficulty box’** if a question **does not apply to you**.

| ***During the past month:*** | No difficulty | A little  difficulty | Quite a bit of difficulty | Very much  difficulty |
| --- | --- | --- | --- | --- |
| 1. Have you had any difficulty maintaining your independence? |  |  |  |  |
| 1. Have you had any difficulty in carrying out your domestic chores? (e.g. cleaning, gardening, cooking, shopping) |  |  |  |  |
| 1. Have you had any difficulty with managing your own personal care? (e.g. bathing, dressing, washing) |  |  |  |  |
| 1. Have you had any difficulty with looking after those who depend on you? (e.g. children, dependent adults, pets) |  |  |  |  |
| 1. Have any of those close to you (e.g. partner, children, parents) had any difficulty with the support available to them? |  |  |  |  |
| 1. Have you had any difficulties with benefits? **(e.g. Statutory Sick Pay, Personal Independence Payments, Attendance Allowance, Universal Credit)** |  |  |  |  |
| 1. Have you had any financial difficulties? |  |  |  |  |
| 1. Have you had any difficulties with financial services? (e.g. loans, mortgages, pensions, insurance) |  |  |  |  |
| 1. Have you had any difficulty concerning your work? (or education if you are a student) |  |  |  |  |
| 1. Have you had any difficulty with planning for your own or your family’s future? (e.g. care of dependents, legal issues, business affairs) |  |  |  |  |
| 1. Have you had any difficulty with communicating with those closest to you? (e.g. partner, children ,parents) |  |  |  |  |
| ***During the past month:*** | No difficulty | A little  difficulty | Quite a bit of difficulty | Very much  difficulty |
| 1. Have you had any difficulty with communicating with others? (e.g. friends, neighbours, colleagues, dates) |  |  |  |  |
| 1. Have you had any difficulty concerning plans to have a family? |  |  |  |  |
| 1. Have you had any difficulty concerning your appearance or body image? |  |  |  |  |
| 1. Have you felt isolated? |  |  |  |  |
| 1. Have you had any difficulty with getting around? (e.g. transport, car parking, your mobility) |  |  |  |  |
| 1. Have you had any difficulty in carrying out your recreational activities (e.g. hobbies, pastimes, social pursuits) |  |  |  |  |
| 1. Have you had any difficulty with your plans to travel or take a holiday? |  |  |  |  |

1. In the **past week**, on how many days have you done a total of 30 minutes or more of physical activity, which was enough to raise your heart rate?

*(This may include sport, exercise and brisk walking or cycling for recreation or to get to and from places,* ***but should not include housework or physical activity that is part of your job.)***

*Please tick* ***one*** *box.*

**None I day 2 days 3 days 4 days 5 days 6 days 7 days**

**Please add anything else you would like to tell us about how your prostate cancer has had an impact on your everyday life.**

Section Five: Your emotional wellbeing

Below are some statements about feelings and thoughts. Please tick the box on each line that best describes your experience of each over **the last 2 weeks.**

| **STATEMENTS** | **None of the time** | **Rarely** | **Some of the time** | **Often** | **All of the time** |
| --- | --- | --- | --- | --- | --- |
| 1. I’ve been feeling optimistic about the future |  |  |  |  |  |
| 1. I’ve been feeling useful |  |  |  |  |  |
| 1. I’ve been feeling relaxed |  |  |  |  |  |
| 1. I’ve been dealing with problems well |  |  |  |  |  |
| 1. I’ve been thinking clearly |  |  |  |  |  |
| 1. I’ve been feeling close to other people |  |  |  |  |  |
| 1. I’ve been able to make up my own mind about things |  |  |  |  |  |

The following questions ask about how you have been feeling during the **past 30 days**.

For each question, please tick the box on each line that best describes how often you had this feeling.

**During the past 30 days, about how often did you feel…**

|  | All of the time | Most of the time | Some of the time | A little of the time | None of the time |
| --- | --- | --- | --- | --- | --- |
| 57. …nervous? |  |  |  |  |  |
| 58. …hopeless? |  |  |  |  |  |
| 59. …restless or fidgety? |  |  |  |  |  |
| 60. …so depressed that nothing could cheer you up? |  |  |  |  |  |
| 61. …that everything was an effort? |  |  |  |  |  |
| 62. …worthless? |  |  |  |  |  |

**Please add anything else you would like to tell us about how your prostate cancer has had an impact on your emotional well-being.**

|  |  |  |  |  |
| --- | --- | --- | --- | --- |

Section Six: Looking to the future

Even if you are now free from prostate cancer please complete this section.

Please read the statements carefully and tick your responses to them. *Please tick* ***one*** *box on each line.* **If a question does not apply to you please leave it blank**.

|  | Strongly agree | Agree | Disagree | Strongly disagree |
| --- | --- | --- | --- | --- |
| 1. I am capable of coping with my prostate cancer |  |  |  |  |
| 1. I have all the information I need to manage my prostate cancer |  |  |  |  |
| 1. I am capable of helping health professionals reach decisions related to my prostate cancer |  |  |  |  |
| 1. My family are very supportive |  |  |  |  |
| 1. I need the support of my family and friends |  |  |  |  |
| 1. My family and friends still rely on me |  |  |  |  |
| 1. I can adapt to the changes in my lifestyle |  |  |  |  |
| 1. Health professionals are happy to include me in decisions related to my prostate cancer |  |  |  |  |
| 1. I want my family and friends to continue to rely on me |  |  |  |  |
| **71.** My friends are always supportive |  |  |  |  |
| **72.**  I still feel useful in my daily life |  |  |  |  |
| **73.** My spiritual beliefs help me cope with my prostate cancer |  |  |  |  |
| **74.** I accept that I have to change my lifestyle |  |  |  |  |
| **75.** Complementary therapies help me cope with my prostate cancer |  |  |  |  |
| **76.** I have a lot of confidence in my local GP |  |  |  |  |
| **77.** How much of an impact has prostate cancer had on your life? | No impact | A little impact | Quite a bit of impact | Very much impact |
|  |  |  |  |  |

**What have been the most important issues that you have faced since your prostate cancer diagnosis?**

Section Seven: Questions about you

| 1. How old are you (in years)? | |  |  |
| --- | --- | --- | --- |
| 1. What is your **legal** marital status? *Please tick* ***one*** *box.* | | | |
|  | Married | | |
|  | In civil partnership | | |
|  | Separated | | |
|  | Divorced/dissolved civil partnership | | |
|  | Widowed/surviving partner from civil partnership | | |
|  | Single (never married/never in civil partnership) | | |
|  | Other | | |
|  | | | |
| 1. What was your employment status before your diagnosis of prostate cancer? *Please tick* ***one*** *box.* | | | |
|  | Full time employment | | |
|  | Part time employment | | |
|  | Self employed | | |
|  | Looking after family/home | | |
|  | Retired | | |
|  | Unemployed, seeking work | | |
|  | Unemployed, unable to work for health reasons | | |
|  | Other | | |

|  | |
| --- | --- |
| 1. What is your employment status currently? *If on sick leave answer in relation to your usual employment status. Please tick* ***one*** *box.* | |
|  | Full time employment |
|  | Part time employment |
|  | Self employed |
|  | Looking after family/home |
|  | Retired |
|  | Unemployed, seeking work |
|  | Unemployed, unable to work for health reasons |
|  | Other |
|  | |
| 1. To which of these ethnic groups would you say you belong? *Please tick* ***one*** *box.* | |
| White | |
|  | English/Welsh/Scottish/Northern Irish/British |
|  | Irish |
|  | Gypsy or Irish Traveller |
|  | Any other White background |
| Mixed/Multiple ethnic groups | |
|  | White and Black Caribbean |
|  | White and Black African |
|  | White and Asian |
|  | Any other Mixed/multiple ethnic background |
| Asian / British Asian | |
|  | Indian |
|  | Pakistani |
|  | Bangladeshi |
|  | Chinese |
|  | Any other Asian background |
| Black/African/Caribbean/Black British | |
|  | Black African |
|  | Black Caribbean |
|  | Any other Black / African / Caribbean background |
| Other ethnic group | |
|  | Arab |
|  | Any other ethnic group |
|  | |
| 1. Do you consider yourself… *Please tick* ***one*** *box.* | |
|  | Heterosexual / straight |
|  | Homosexual / gay |
|  | Bisexual |
|  | Don’t know |
|  | Prefer not to answer |
|  | |
| 1. Which, if any, of the following conditions do you have? *Please tick* ***all*** *the boxes that apply.* | |
|  | A heart condition |
|  | Angina |
|  | High blood pressure |
|  | Asthma or other chronic chest problem |
|  | Liver disease |
|  | Problems with your stomach, bowels or gallbladder |
|  | Problems with your pancreas |
|  | Kidney disease |
|  | Diabetes |
|  | Stroke |
|  | Alzheimer’s disease or dementia |
|  | Epilepsy |
|  | Other long standing neurological problem |
|  | A diagnosis of arthritis |
|  | |
| 1. How tall are you? ……feet…….inches OR ……centimetres Don’t know | |
| 1. How much do you weigh? …….stone…...pounds OR ….kilograms… grams Don’t know | |
|  | |
| 1. Have you ever in your lifetime seen a health care professional (such as a GP, psychiatrist, psychologist, social worker, counsellor, psychotherapist, mental health nurse, or any other such professional) for problems with your emotions or nerves or your use of alcohol or drugs? | |
| Yes No | |
| 1. Do you look after, or give any help or support (not part of your paid employment) to family members, friends, neighbours or others because of either:  - Long term physical or mental health disability, or - Problems relating to old age | |
| Yes No | |

**Is there anything else you would like to tell us about what life has been like for you following your prostate cancer diagnosis?**

Please would you tell us who filled in this survey? *Please tick* ***one*** *box.*

The person to whom this survey was sent

A representative of the person to whom this survey was sent

(e.g. partner, family member, friend)

**Follow-up survey**

Thank you for completing this survey. We will be **contacting you again in a years time** for the follow-up survey. If you decide at that time you would rather not complete the survey again there is no obligation to do so.

**Follow-up interview**

As part of this work we are going to be interviewing a small number of men and some partners /spouses to gain a better understanding of their experience. This will only involve men and their partners / spouses who indicate they are interested in being interviewed. We would normally only be able to interview either the participant or their partner/spouse. We will not be interviewing couples together.

If you or your partner/spouse is interested in being involved in the interview, please tick the relevant boxes below.

I am interested in being interviewed for this work

My partner / spouse is interested in being interviewed for this work

We are sorry we will not be able to contact all those who would like to participate in the interview, but we will make sure that we get the views of a varied group. If you are one of the group we would like to interview we will write to you explaining how to get in touch with us to find out more about the interview. If you are still interested, we would arrange a time for the interview. If you think your partner/spouse would like to be interviewed, we would follow a similar process by making the initial contact with you by letter.

**If you would like to know more about this study then please visit our website: http://www.lifeafterprostatecancerdiagnosis.com/**

*We very much appreciate the time and thought you have put into completing this survey. If reflecting on your situation has caused anxiety or uncertainty in any way, please do not hesitate to contact your specialist cancer nurse or call one of the specialist nurses on Prostate Cancer UK’s Confidential Help Line.*

*Prostate Cancer UK’s Specialist Nurse Telephone Line:* ***0800 074 8383***

*(Free from UK landlines)*

*(Monday – Friday 9-6pm, Wednesday 10-9pm).*

*They are there to listen to your concerns, and offer support and helpful information*

You have completed the survey. Thank you for your time.

**Supplementary File 2. Treatment data additional details**

In the overall study sample, a much higher than expected proportion (3.4%) reporting having High Intensity Focused Ultrasound (HIFU), indicating some confusion with this terminology. This group was excluded from analyses by treatment type, as were men reporting having radiotherapy who did not know which type (4.6%) and those who were unsure which type of follow-up they were receiving (2.1%).

In the group of men with stage I, II or III disease analysed for this paper, the proportions were as follows: 3.5% reporting having High Intensity Focused Ultrasound (HIFU), 4.2% reported unknown radiotherapy and 1.9% reported unknown follow-up.

**Supplementary Table 1: Comparison of responders and non-responders**

| **Characteristic** |  | **Non-responder** | **Responder** | **Total** | **Response rate** |
| --- | --- | --- | --- | --- | --- |
| **Age at diagnosis*** | <55 years | 1,293 | 1,392 | 2,685 | ***51.8%*** |
|  | 55-64 years | 5,013 | 8,529 | 13,542 | ***63.0%*** |
|  | 65-74 years | 8,886 | 16,992 | 25,878 | ***65.7%*** |
|  | 75-84 years | 6,407 | 8,094 | 14,501 | ***55.8%*** |
|  | 85+ years | 1,385 | 810 | 2,195 | ***36.9%*** |
|  | Unknown | 123 | 6 | 129 |  |
|  | Total | 23,107 | 35,823 | 58,930 | ***60.8%*** |
| **Ethnic group*^†^** | White | 17,493 | 28,705 | 46,198 | ***62.1%*** |
|  | Mixed | 121 | 78 | 199 | ***39.2%*** |
|  | Asian | 536 | 333 | 869 | ***38.3%*** |
|  | Black | 1,040 | 586 | 1,626 | ***36.0%*** |
|  | Other | 210 | 173 | 383 | ***45.2%*** |
|  | Unknown | 481 | 590 | 1,071 | ***55.1%*** |
|  | Total | 19,881 | 30,465 | 50,346 | ***60.5%*** |
| **Stage of disease at diagnosis** | 1 | 7,606 | 10,880 | 18,486 | ***58.9%*** |
|  | 2 | 4,776 | 8,719 | 13,495 | ***64.6%*** |
|  | 3 | 3,761 | 7,209 | 10,970 | ***65.7%*** |
|  | 4 | 2,825 | 3,925 | 6,750 | ***58.1%*** |
|  | Unknown | 4,139 | 5,090 | 9,229 | ***55.2%*** |
|  | Total | 23,107 | 35,823 | 58,930 | ***60.8%*** |
| **Deprivation quintile** | 1 - least deprived | 4,446 | 9,408 | 13,854 | ***67.9%*** |
|  | 2 | 4,978 | 9,289 | 14,267 | ***65.1%*** |
|  | 3 | 4,782 | 7,381 | 12,163 | ***60.7%*** |
|  | 4 | 4,401 | 5,266 | 9,667 | ***54.5%*** |
|  | 5 - most deprived | 3,881 | 3,620 | 7,501 | ***48.3%*** |
|  | Unknown | 619 | 859 | 1,478 | ***58.1%*** |
|  | Total | 23,107 | 35,823 | 58,930 | ***60.8%*** |

*This table shows registry-collected age at diagnosis and ethnic group for comparison with the non-responders. Presented are aggregated data only for the non-responders and therefore this table also includes the men diagnosed with stage IV disease who were invited to participate in the Life After Prostate Cancer Diagnosis study.

**^†^**Registry-collected ethnic group data only available for England.

**Supplementary Table 2. Sociodemographic and clinical characteristics by level of decision regret**

| **Characteristics** | | **All DRS responders** | | **No regret** | | **Mild regret** | | **Moderate/ severe regret** | |  |
| --- | --- | --- | --- | --- | --- | --- | --- | --- | --- | --- |
|  | | **N** | **%** | **N** | **%** | **N** | **%** | **N** | **%** | **p** |
|  |  | **17,193** | **100** | **6,297** | **36.6** | **7,450** | **43.3** | **3,446** | **20.0** |  |
| **Age** | <60 years | 2,640 | 15.4 | 1,036 | 39.2 | 1,047 | 39.7 | 557 | 21.1 | <0.001 |
|  | 60-69 years | 8,217 | 47.8 | 3,209 | 39.1 | 3,466 | 42.2 | 1,542 | 18.8 |  |
|  | 70-79 years | 5,527 | 32.1 | 1,856 | 33.6 | 2,542 | 46.0 | 1,129 | 20.4 |  |
|  | 80+ years | 809 | 4.7 | 196 | 24.2 | 395 | 48.8 | 218 | 26.9 |  |
| **Ethnicity** | White | 16,402 | 97.4 | 6,062 | 37.0 | 7,109 | 43.3 | 3,231 | 19.7 | <0.001 |
|  | Non-white | 438 | 2.6 | 119 | 27.2 | 198 | 45.2 | 121 | 27.6 |  |
| **Marital status** | Married/Civil Partner | 13,937 | 81.7 | 5,253 | 37.7 | 6,070 | 43.6 | 2,614 | 18.8 | <0.001 |
|  | Separated/Divorced | 1,238 | 7.3 | 390 | 31.5 | 518 | 41.8 | 330 | 26.7 |  |
|  | Widowed | 1,049 | 6.1 | 334 | 31.8 | 465 | 44.3 | 250 | 23.8 |  |
|  | Single | 609 | 3.6 | 200 | 32.8 | 254 | 41.7 | 155 | 25.5 |  |
|  | Other | 232 | 1.4 | 83 | 35.8 | 91 | 39.2 | 58 | 25.0 |  |
| **No. other LTCs** | 0 | 5,141 | 29.9 | 2,095 | 40.8 | 2,152 | 41.9 | 894 | 17.4 | <0.001 |
|  | 1 | 6,211 | 36.1 | 2,359 | 38.0 | 2,710 | 43.6 | 1,142 | 18.4 |  |
|  | 2 | 3,256 | 18.9 | 1,098 | 33.7 | 1,444 | 44.3 | 714 | 21.9 |  |
|  | 3 | 1,429 | 8.3 | 430 | 30.1 | 635 | 44.4 | 364 | 25.5 |  |
|  | 4+ | 1,156 | 6.7 | 315 | 27.2 | 509 | 44.0 | 332 | 28.7 |  |
| **Socio-economic deprivation quintile** | 1 least deprived | 4,820 | 28.6 | 1,874 | 38.9 | 2,077 | 43.1 | 869 | 18.0 | <0.001 |
|  | 2 | 4,663 | 27.7 | 1,753 | 37.6 | 2,014 | 43.2 | 896 | 19.2 |  |
|  | 3 | 3,526 | 20.9 | 1,271 | 36.0 | 1,529 | 43.4 | 726 | 20.6 |  |
|  | 4 | 2,375 | 14.1 | 813 | 34.2 | 1,051 | 44.3 | 511 | 21.5 |  |
|  | 5 most deprived | 1,466 | 8.7 | 458 | 31.2 | 634 | 43.2 | 374 | 25.5 |  |
| **Stage at diagnosis** | I/II | 12,748 | 74.1 | 4,759 | 37.3 | 5,468 | 42.9 | 2,521 | 19.8 | 0.01 |
|  | III | 4,445 | 25.9 | 1,538 | 34.6 | 1,982 | 44.6 | 925 | 20.8 |  |
| **Treatment** | Active surveillance/Watchful waiting | 3,028 | 17.6 | 1,151 | 38.0 | 1,309 | 43.2 | 568 | 18.8 | <0.001 |
|  | Surgery alone | 4,824 | 28.1 | 2,027 | 42.0 | 1,908 | 39.6 | 889 | 18.4 |  |
|  | EBRT alone | 1,435 | 8.3 | 507 | 35.3 | 648 | 45.2 | 280 | 19.5 |  |
|  | Brachytherapy alone | 825 | 4.8 | 383 | 46.4 | 318 | 38.5 | 124 | 15.0 |  |
|  | ADT alone | 969 | 5.6 | 224 | 23.1 | 478 | 49.3 | 267 | 27.6 |  |
|  | EBRT & ADT | 4,839 | 28.1 | 1,572 | 32.5 | 2,252 | 46.5 | 1,015 | 21.0 |  |
|  | Surgery & EBRT/ADT | 1,273 | 7.4 | 433 | 34.0 | 537 | 42.2 | 303 | 23.8 |  |

LTCs: long term conditions; EBRT: External beam radiotherapy; ADT: Androgen deprivation therapy; DRS: Decision regret scale.

Data were missing as follows: Ethnicity: 353 (2.1%) records; Marital status: 128 (0.8%) records; Socio-economic deprivation quintile: 343 (2.0%) records.

**Supplementary Table 3. Completeness of each item within the decision regret scale**

| **Item** | **Strongly agree** | **Agree** | **Neither agree or disagree** | **Disagree** | **Strongly disagree** | **Missing** | **Total** |
| --- | --- | --- | --- | --- | --- | --- | --- |
| It was the right decision | 13,394 | 6,983 | 1,200 | 173 | 59 | 869 | 22,678 |
|  | 59.1% | 30.8% | 5.3% | 0.8% | 0.3% | 3.8% | 100.0 |
| I regret the choice that was made | 466 | 865 | 1,608 | 4,852 | 10,073 | 4,814 | 22,678 |
|  | 2.1% | 3.8% | 7.1% | 21.4% | 44.4% | 21.2% | 100.0 |
| I would go for the same choice again if I had to do it over again | 11,088 | 5,934 | 1,574 | 466 | 280 | 3,336 | 22,678 |
|  | 48.9% | 26.2% | 6.9% | 2.1% | 1.2% | 14.7% | 100.0 |
| The choice did me a lot of harm | 298 | 773 | 2,023 | 5,157 | 9,747 | 4,680 | 22,678 |
|  | 1.3% | 3.4% | 8.9% | 22.7% | 43.0% | 20.6% | 100.0 |
| The decision was a wise one | 10,853 | 6,156 | 1,798 | 265 | 222 | 3,384 | 22,678 |
|  | 47.9% | 27.1% | 7.9% | 1.2% | 1.0% | 14.9% | 100.0 |

**Supplementary Table 4. Characteristics of non-responders to the decision regret scale**

| **Characteristics** |  | **Non-responders to DRS (n)** | **Total responders (n)** | **Non-responders to DRS (%)** |  |
| --- | --- | --- | --- | --- | --- |
|  |  | 5,165 | 22,358 | 23.1 |  |
| **Age** | <60 years | 297 | 2,937 | 10.1 | <.001 |
|  | 60-69 years | 1,864 | 10,081 | 18.5 |  |
|  | 70-79 years | 2,419 | 7,946 | 30.4 |  |
|  | 80+ years | 585 | 1,394 | 42.0 |  |
| **Ethnicity** | White | 4,730 | 21,132 | 22.4 | <.001 |
|  | Non-white | 183 | 621 | 29.5 |  |
|  | Unknown | 252 | 605 | 41.7 |  |
| **Marital status** | Married/Civil Partner | 3,963 | 17,900 | 22.1 | <.001 |
|  | Separated/Divorced | 368 | 1,606 | 22.9 |  |
|  | Widowed | 467 | 1,516 | 30.8 |  |
|  | Single | 203 | 812 | 25.0 |  |
|  | Other | 61 | 293 | 20.8 |  |
|  | Unknown | 103 | 231 | 44.6 |  |
| **No. other LTCs** | 0 | 1,449 | 6,590 | 22.0 | <.001 |
|  | 1 | 1,780 | 7,991 | 22.3 |  |
|  | 2 | 1,084 | 4,340 | 25.0 |  |
|  | 3 | 472 | 1,901 | 24.8 |  |
|  | 4+ | 380 | 1,536 | 24.7 |  |
| **Socio-economic deprivation quintile** | 1 (least deprived) | 1,112 | 5,932 | 18.7 | <.001 |
|  | 2 | 1,352 | 6,015 | 22.5 |  |
|  | 3 | 1,112 | 4,638 | 24.0 |  |
|  | 4 | 831 | 3,206 | 25.9 |  |
|  | 5 (most deprived) | 653 | 2,119 | 30.8 |  |
|  | Unknown | 105 | 448 | 23.4 |  |
| **Stage at diagnosis** | I/II | 3,823 | 16,571 | 23.1 | 0.85 |
|  | III | 1,342 | 5,787 | 23.2 |  |
| **Treatment** | Active surveillance/watchful waiting | 1,140 | 4,168 | 27.4 | <.001 |
|  | Surgery alone | 1,105 | 5,929 | 18.6 |  |
|  | EBRT alone | 671 | 2,106 | 31.9 |  |
|  | Brachytherapy alone | 177 | 1,002 | 17.7 |  |
|  | ADT alone | 483 | 1,452 | 33.3 |  |
|  | EBRT & ADT | 1,208 | 6,047 | 20.0 |  |
|  | Surgery & EBRT/ADT | 381 | 1,654 | 23.0 |  |

EBRT: External beam radiotherapy; ADT: Androgen deprivation therapy.

**Supplementary Table 5. Multivariable ordinal regression analysis of the association between involvement in decision-making and decision regret**

| **Response to involvement item^1^** | **n** | **%** | **Adjusted OR*** | **95% CI** |
| --- | --- | --- | --- | --- |
| Yes, definitely | 12,574 | 73.9 | 1.00 |  |
| Yes, to some extent | 3077 | 18.1 | 4.63 | 4.27-5.02 |
| No, views not taken into account | 545 | 3.2 | 6.42 | 5.39-7.64 |
| Not sure or can't remember | 829 | 4.9 | 4.61 | 4.00-5.32 |

^1^Do you think your views were taken into account when the team of doctors and nurses caring for you were discussing which treatment you should have?

*The ordinal regression results compare mild/moderate/severe regret vs no regret and moderate/severe regret vs mild/no regret). Adjusted for age, ethnicity, marital status, number of other long-term conditions, socio-economic deprivation quintile and stage of disease.

OR: Odds ratio, CI: Confidence Interval

**Supplementary Table 6. STROBE statement: checklist for cross sectional studies**

|  |  |  |  |  |
| --- | --- | --- | --- | --- |
|  |  | **Item No** | **Recommendation** | **Response** |
|  | **Title and abstract** | **1** | (a) Indicate the study’s design with a commonly used term in the title or the abstract | Page 1, 2 |
|  |  |  | (b) Provide in the abstract an informative and balanced summary of what was done and what was found | Page 2 |
|  | **Introduction** |  |  |  |
|  | Background /rationale | 2 | Explain the scientific background and rationale for the investigation being reported | Page 3-4 |
|  | Objectives | 3 | State specific objectives, including any prespecified hypotheses | Page 4 |
|  | **Methods** |  |  |  |
|  | Study design | 4 | Present key elements of study design early in the paper | Page 4-5 |
|  | Setting | 5 | Describe the setting, locations, and relevant dates, including periods of recruitment,exposure, follow-up, and data collection | Page 5 |
|  | Participants | 6 | (a) Give the eligibility criteria, and the sources and methods of selection of participants | Page 4 |
|  | Variables | 7 | Clearly define all outcomes, exposures, predictors, potential confounders, and effect modifiers. Give diagnostic criteria, if applicable | Page 5-6 |
|  | Data sources / measurement | 8* | For each variable of interest, give sources of data and details of methods of assessment (measurement). Describe comparability of assessment methods if there is more than one group | Page 5-6 |
|  | Bias | 9 | Describe any efforts to address potential sources of bias | Page 4-7 |
|  | Study size | 10 | Explain how the study size was arrived at | Page 4 |
|  | Quantitative variables | 11 | Explain how quantitative variables were handled in the analyses. If applicable, describe which groupings were chosen and why | Page 6 |
|  | Statistical methods | 12 | (a) Describe all statistical methods, including those used to control for confounding | Page 6-7 |
|  |  |  | (b) Describe any methods used to examine subgroups and interactions | N/A |
|  |  |  | (c) Explain how missing data were addressed | Page 7-9 |
|  |  |  | (d) If applicable, describe analytical methods taking account of sampling strategy | N/A |
|  |  |  | (e) Describe any sensitivity analyses | N/A |
|  | **Results** |  |  |  |
|  | Participants | 13* | (a) Report numbers of individuals at each stage of study—eg numbers potentially eligible, examined for eligibility, confirmed eligible, included in the study, completing follow-up, and analysed | Supplementary figure 4 |
|  |  |  | (b) Give reasons for non-participation at each stage |  |
|  |  |  | (c) Consider use of a flow diagram |  |
|  | Descriptive data | 14* | (a) Give characteristics of study participants (eg demographic, clinical, social) and information on exposures and potential confounders | Page 7, Supplementary Table 2 |
|  |  |  | (b) Indicate number of participants with missing data for each variable of interest | Supplementary Table 3-4 |
|  | Outcome data | 15* | Report numbers of outcome events or summary measures | Page 8-10 |
|  | Main results | 16 | (a) Give unadjusted estimates and, if applicable, confounder-adjusted estimates and their precision (eg, 95% confidence interval). Make clear which confounders were adjusted for and why they were included | Page 8-10 |
|  |  |  | (b) Report category boundaries when continuous variables were categorized | Page 7; Supplementary Table 2. |
|  |  |  | (c) If relevant, consider translating estimates of relative risk into absolute risk for a meaningful time period | N/A |
|  | Other analyses | 17 | Report other analyses done—eg analyses of subgroups and interactions, and sensitivity analyses | N/A |
|  | **Discussion** |  |  |  |
|  | Key results | 18 | Summarise key results with reference to study objectives | Page 10 |
|  | Limitations | 19 | Discuss limitations of the study, taking into account sources of potential bias or imprecision. Discuss both direction and magnitude of any potential bias | Page 11 |
|  | Interpretation | 20 | Give a cautious overall interpretation of results considering objectives, limitations, multiplicity of analyses, results from similar studies, and other relevant evidence | Page 11-12 |
|  | Generalisability | 21 | Discuss the generalisability (external validity) of the study results | Page 11-12 |
|  | **Other information** |  |  |  |
|  | Funding | 21 | Give the source of funding and the role of the funders for the present study and, if applicable, for the original study on which the present article is based | Page 14 |
|  | *Give information separately for exposed and unexposed groups. | | |  |
